# QTL Analysis of Spike Morphological Traits and Plant Height in Winter Wheat (*Triticum aestivum* L.) Using a High-Density SNP and SSR-Based Linkage Map

**DOI:** 10.3389/fpls.2016.01617

**Published:** 2016-11-07

**Authors:** Huijie Zhai, Zhiyu Feng, Jiang Li, Xinye Liu, Shihe Xiao, Zhongfu Ni, Qixin Sun

**Affiliations:** ^1^State Key Laboratory for Agrobiotechnology, Key Laboratory of Crop Heterosis and Utilization, Beijing Key Laboratory of Crop Genetic Improvement, China Agricultural UniversityBeijing, China; ^2^National Plant Gene Research CentreBeijing, China; ^3^Institute of Crop Science, Chinese Academy of Agricultural SciencesBeijing, China

**Keywords:** SNP, SSR, QTL, spike morphology, plant height, wheat

## Abstract

Wheat yield can be enhanced by modifying the spike morphology and the plant height. In this study, a population of 191 F_9_ recombinant inbred lines (RILs) was developed from a cross between two winter cultivars Yumai 8679 and Jing 411. A dense genetic linkage map with 10,816 markers was constructed by incorporating single nucleotide polymorphism (SNP) and simple sequence repeat (SSR) marker information. Five spike morphological traits and plant height were evaluated under nine environments for the RILs and parental lines, and the number of detected environmentally stable QTLs were 18 and three, respectively. The 1RS/1BL (rye) translocation increased both spike length and spikelet number with constant spikelet compactness. The *QPht.cau-2D.1* was identical to gene *Rht8*, which decreased spike length without modifying spikelet number. Notably, four novel QTLs locating on chromosomes 1AS (*QSc.cau-1A.1*), 2DS (*QSc.cau-2D.1*), and 7BS (*QSl.cau-7B.1* and *QSl.cau-7B.2*) were firstly identified in this study, which provide further insights into the genetic factors that shaped the spike morphology in wheat. Moreover, SNP markers tightly linked to previously reported QTLs will eventually facilitate future studies including their positional cloning or marker-assisted selection.

## Introduction

Wheat is the leading food crop produced, consumed, and traded worldwide today, and China is the largest wheat producer and consumer in the world (Wang et al., 2009). Since wheat spike is an important reproductive organ, a number of studies have demonstrated that spike morphological traits (e.g., spike length, SL) are positively correlated with grain yield (Kumar et al., 2007) and yield components (e.g., thousand grain weight; Wu et al., 2012; Gao et al., 2015). Therefore, genes or quantitative trait loci (QTLs) associated with spike morphological traits are of interest for breeding purposes.

*Q, C*, and *S* are three well-known loci that have been recruited for the domestication of wheat spike morphological traits (Faris et al., 2014b). The *Q* locus pleiotropically affects a wide range of traits, including plant height, spike length, and rachis fragility (Simons et al., 2006). The *C* locus affects spike morphology, grain size, shape, and number, while the *S* locus determines whether a spike has round seeds and glumes (Salina et al., 2000; Johnson et al., 2008). However, various spike morphological traits among modern cultivars are unlikely contributed by these three major genes, because all common wheat accessions have the universal genotype (*QcS*; Faris et al., 2014b).

Three groups of genes, vernalization (*Vrn*), photoperiod (*Ppd*), and earliness *per-se* (*Eps*), controlling the life-cycle duration in wheat are important for freezing resistance, heading time, and yield component generation (Wang L. et al., 2015). The vernalization insensitive alleles of *Vrn-1* (*Vrn-A1, Vrn-B1*, and *Vrn-D1*) shorten both the vegetative and the reproductive stages (Snape et al., 2001) and have considerable impact on spike morphological traits (Kato et al., 2000). Photoperiod insensitive alleles of *Ppd-1* (*Ppd-A1a, Ppd-B1a*, and *Ppd-D1a*) bring forward the time of terminal spikelet, and hence reduce the spikelet number (Snape et al., 2001). Compared with *Vrn* and *Ppd* genes, *Eps* genes have less evident effects on life-cycle duration but are also involved in spike development. For example, the *Eps-A*^*m*^*1* gene from diploid wheat *Triticum monococcum* affects heading time, spike development, and spikelet number (Faricelli et al., 2010).

By conferring insensitivity to specific kind of plant hormones, reduced height (*Rht*) or dwarfing genes can increase grain yield and are always involved in manipulation of spike morphology in wheat. *Rht-B1, Rht-D1*, and *Rht8* are three most commonly adopted dwarfing genes worldwide. *Rht-B1* and *Rht-D1* are two gibberellins (GAs) insensitive dwarfing genes, and have a profound impact on stem elongation and vegetative dry-matter accumulation (Youssefian et al., 1992). Compared with tall plants, semi-dwarfed plants have a greater portion of assimilate allocated to the developing spikes, which results in improved spikelet fertility and increased grain number per spike (Youssefian et al., 1992; Flintham et al., 1997). *Rht8* is a brassinosteroids (BRs) insensitive dwarfing gene located on chromosome 2DS (Korzun et al., 1998; Gasperini et al., 2012). Introgression lines carrying the semi-dwarfing allele (*Rht8c*) have shortened spikes with constant spikelet number, resulting in semi-compacted spike morphology (Kowalski et al., 2016). Moreover, coincidence of QTLs between traits obtained by means of QTL analysis can also provide a clear understanding of the genetic relationship between plant height (PHT) and spike morphological traits.

Over the past two decades, the successful application of quantitative-genetic methodology has facilitated identification of numerous QTLs for spike morphological traits and plant height on all 21 wheat chromosomes (Cui et al., 2012). Notably, consistent QTLs across multiple genetic backgrounds have been identified on chromosomes 1B, 2D, 4B, 5A, and 7A (Jantasuriyarat et al., 2004; Kumar et al., 2007; Cui et al., 2012; Xu et al., 2014). However, to our knowledge, most studies mapping QTLs for spike morphological traits and plant height used low-density genetic maps and mapped QTLs within relatively large confidence intervals, hence hindering their possible applications in wheat breeding programs. Here, we report the construction of high-density genetic linkage map using SNP and SSR markers, and the identification of environmentally stable QTLs associated with spike morphology and plant height using the Yumai 8679 (Y8679)/Jing 411 (J411) recombinant inbred line (RIL) population. The results provide further insights into the genetic factors that shaped the spike morphology in wheat.

## Materials and methods

### Plant materials and field trials

**Figure 1:**
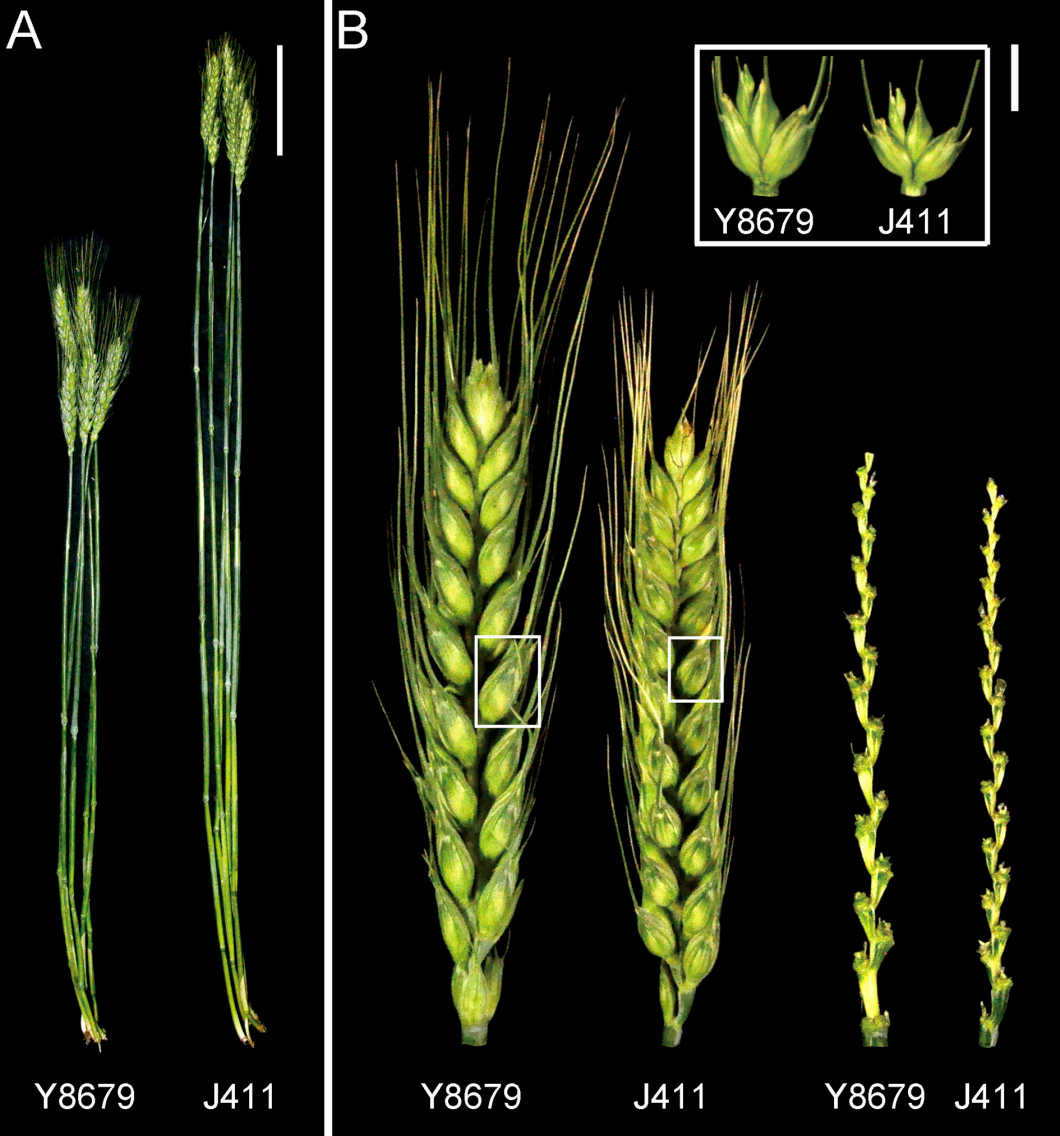
**Culm and spike morphology of the parental lines grown in Beijing (2015–2016 growing season)**. **(A)** Main tillers of Y8679 (left) and J411 (right). The bar represents 10 cm. **(B)** Spikes, spikelets, and rachises of parent Y8679 (left) and parent J411 (right). The bar represents 1 cm.

A population comprising of 191 RILs was developed by crossing two winter cultivars (Y8679 and J411) and advanced to the F_9_ generation by single seed descent method. Y8679 has higher spike length and J411 has higher spikelet number per spike (Figure 1). Generations F_9_ to F_12_ of the RIL population were included in this study (Table S1). The RIL population and the two parents were grown at four locations (Beijing, Anhui, Shaanxi, and Hebei) during four growing seasons from 2010–2011 to 2014–2015, providing data for nine environments (Table S1). These four locations are representative areas of two main wheat production zones (Northern Winter Wheat Zone and Yellow and Huai River Valleys Facultative Wheat Zone; Figure S1), which produce 68% of total wheat production in China (Wang et al., 2009). Location-year information and climate data across the whole life cycle are presented in Figure S1 and Table S1. Weather conditions for the same locations across years were generally similar. Environments at lower latitudes had higher monthly average maximum and minimum temperatures prior to anthesis.

In each environment, the 191 RILs and the two parents were planted in a randomized complete block design with three replicates. Each plot contained two rows that were 2 m long and 0.3 m apart with a sowing rate at 30 seeds per row. All fields were well-watered by both rainfall and broad irrigation. Other management of field trials was in accordance with local standard practices.

### Phenotypic evaluation and statistical analysis

At maturity, 10 representative plants from each genotype were used for phenotypic evaluation. All data were collected from the main tillers. PHT was determined by measuring from the ground to the tip of the spike excluding awns. Spike length (SL) was measured from the base of the rachis to the tip of the terminal spikelet excluding the awns. Fertile spikelet number per spike (FSN) and sterile spikelet number per spike (SSN) were determined by counting the number of fertile and sterile spikelets. Total spikelet number per spike (TSN) was calculated by summing the values of FSN and SSN. Spikelet compactness (SC) was calculated by dividing the SL by the TSN.

The statistical and correlation analyses were performed with SPSS version 20.0 (SPSS, Chicago, USA). The Shapiro-Wilk test was performed with R software (V. 3.2.2) to test for departures from normality. The adjusted mean (Best Linear Unbiased Prediction, BLUP) values across evaluated environments were calculated using the PROC MIXED procedure in SAS (SAS Institute Inc., North Carolina, USA). Broad sense heritability ($h_{B}^{2}$) based on a family mean basis was calculated using the PROC GLM procedure in SAS (SAS Institute Inc., North Carolina, USA) based on the following formula: $h_{B}^{2}=\sigma_{g}^{2}/\left(\sigma_{g}^{2}+\sigma_{ge}^{2}/n+\sigma^{2}/nr\right)$, in which $\sigma_{g}^{2}$ is the genotypic effect, σ_*ge*_^2^ is the genotype by environmental effect, σ^2^ is the residual error, *n* is the number of environments and *r* is the number of replicates.

### Genotyping and linkage analyses

The Y8679/J411 RIL population along with the two parents was genotyped using the iSelect 90K array containing 90,000 wheat SNP markers (Wang et al., 2014). Twenty seeds from each genotype were germinated, and young leaves were used for DNA extraction at the seedling stage. SNP genotyping analysis was performed at the Genome Center of the University of California at Davis according to the manufacturer's protocols (Illumina). SNP clustering and genotype calling were performed using GenomeStudio version 2011.1 software (Illumina).

In addition, 215 SSR markers with known chromosome locations were used to help anchor linkage groups into specific chromosomes. Most SSR primers are publicly available at http://wheat.pw.usda.gov/GG2/index.shtml. Several SSR primers designed by our lab can be accessed in Zhai et al. (2015). Parental polymorphism survey and validation of polymorphic SSR markers were conducted using the PCR conditions described by Zhai et al. (2015).

Two 1RS specific markers, *pSaD15* and *pSc20H* (Liu et al., 2008), were used for identification of the RILs with a 1RS/1BL translocation. The primer sequences for the *pSaD15* set are 5′-CCGGCGTGTCGACACCCTGATA-3′ and 5′-CATCCGTGCTCCGTGTGCATC-3′ and an annealing temperature of 60°C was used. The primer sequences for the *pSc20H* set are 5′-GTTGGAAGGGAGCTCGAGCTG-3′ and 5′-GTTGGGCAGAAAGGTCGACATC-3′ and an annealing temperature of 60°C was used.

Genetic linkage maps were constructed with programs RECORD 2.0 (Van Os et al., 2005) and JoinMap 4.0 (Van Ooijen, 2006). Redundant markers with identical segregations were firstly identified and removed using RECORD 2.0. Unique markers were further organized into linkage groups using JoinMap 4.0 with LOD thresholds ranged from 5 to 10. The order of markers within a linkage group was established based on a regression-mapping algorithm (Stam, 1993). Map distances were calculated from recombination frequencies using the Kosambi mapping function (Kosambi, 1943). Removed redundant markers were finally placed beside their kept representatives into the map. The identity, polarity, and centromere positions of linkage groups were determined based on the best blastn hits of the nucleotide sequence flanking the SNP against the Chromosome Survey Sequence (CSS) contigs (Wang et al., 2014).

### Bioinformatics analysis

Mapped SNPs were annotated by comparing flanking sequences with the wheat unigene database (158,028 unigenes) from NCBI (http://www.ncbi.nlm.nih.gov) using the BLASTN program (e-value cutoff ≤ 1e^−10^). Only the best hits were kept. For functional annotation, attached wheat unigenes were selected and compared against the protein sequences (BLASTX e-value cutoff ≤ 1e^−10^) predicted in the rice and *Brachypodium* genomes (International Rice Genome Sequencing Project, 2005; Vogel et al., 2010). Only the best hits were retained.

### QTL analysis

The trait values of three replicates under each environment were averaged and used for QTL analysis. The adjusted mean (BLUP) values for the PHT, SL, FSN, SSN, TSN, and SC of each genotype across the nine environments were used for the combined analysis. The QTLs were scanned with QTL Cartographer version 2.5 (Wang et al., 2012) through composite interval mapping (CIM). In this method, model 6 with forward and backward regression, five markers as cofactors and a 10-cM scanning window was used for the detection of QTLs. Empirical threshold LOD scores for CIM were calculated with 1000 permutations at *P* ≤ 0.05. Confidence intervals were acquired based on positions ± 2 LOD away from the peaks of the likelihood ratios (LRs). The QTLs with overlapping confidence intervals were treated as equivalent. The QTLs were denoted according to McIntosh et al. (2011).

## Results

### Phenotypic evaluation

**Figure 2:**
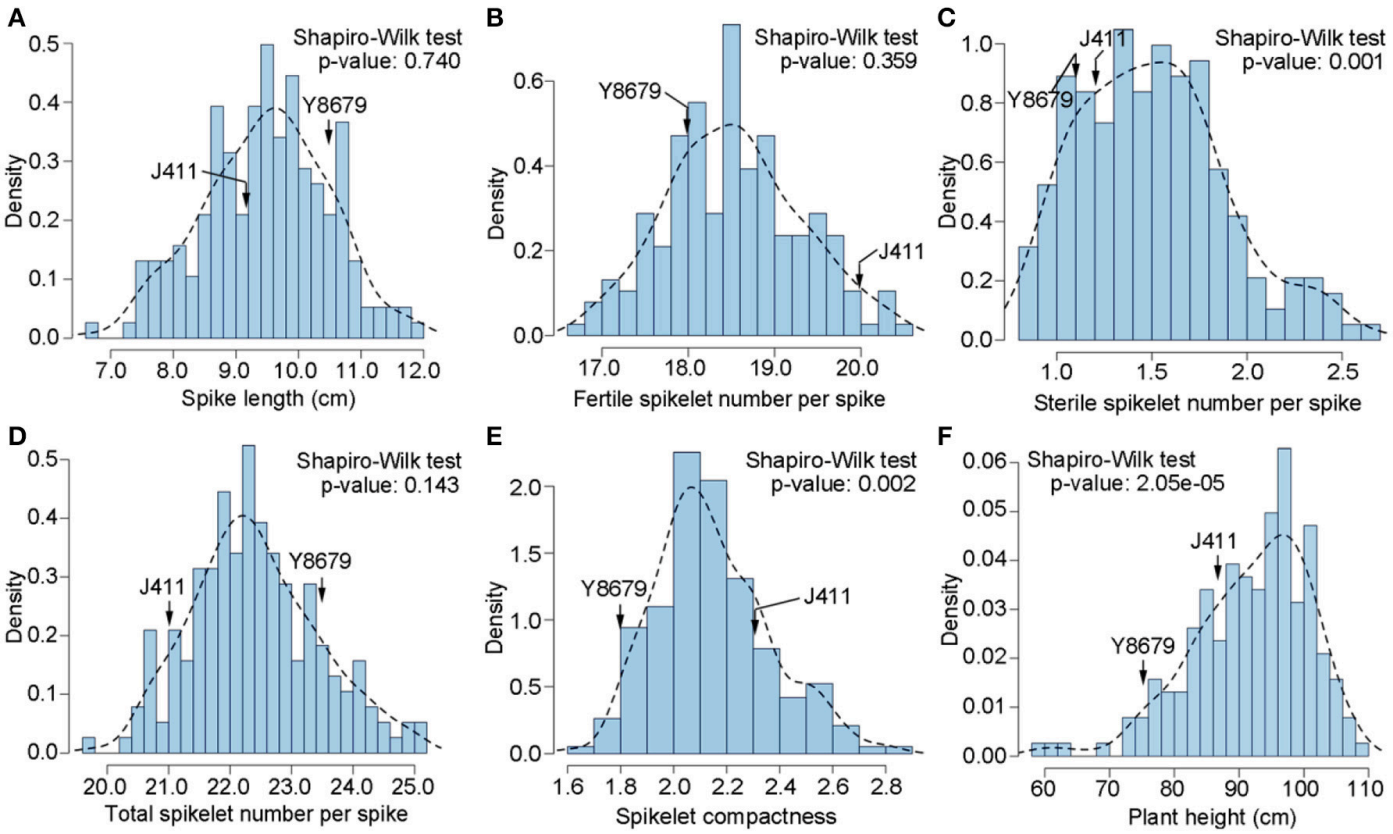
**Histograms of the Y8679/J411 recombinant inbred population for (A) spike length (SL), (B) fertile spikelet number per spike (FSN), (C) sterile spikelet number per spike (SSN), (D) total spikelet number per spike (TSN), (E) spikelet compactness (SC), and (F) plant height (PHT)**.

Parental and population means and ranges for the six traits are listed in Table 1. J411 had higher PHT, FSN, TSN, and SC across environments (Figure 1; Table 1; Appendix C). Conversely, Y8679 had an average SL that was 1.33 cm longer than that of J411 (Table 1). Shapiro-Wilk test for normality and estimation of broad sense heritability based on a family mean basis were conducted for the six investigated traits. SL, FSN, and TSN showed normal distribution, whereas PHT, SSN, and SC departed significantly from normality at the 0.01 significance level (Figure 2). PHT, SL, FSN, TSN, and SC had high heritabilities ($h_{B}^{2}$ > 0.90), and SSN had a relatively lower heritability (${\text{h}}_{B}^{2}$ = 0.84). Phenotypic values in the 191 RILs showed bi-directional transgressive segregation for all traits (Figure 2), suggesting that both parents contributed increasing alleles to these traits. Estimated correlation coefficients among the six traits are listed in Table 2. SL had strong positive correlations with FSN, SSN, TSN, and PHT, and a stronger negative correlation with SC. SC had a strong positive correlation with FSN, and stronger negative correlations with SL and SSN. In addition to a strong positive correlation with SL and SSN, PHT had a weak negative correlation with FSN and a strong negative correlation with SC.

**Table 1:** **Parental and population means, ranges, and broad sense heritabilities for spike length (SL), fertile spikelet number per spike (FSN), sterile spikelet number per spike (SSN), total spikelet number per spike (TSN), spikelet compactness (SC), and plant height (PHT)**.

**Traits**	**Mean**			**RIL population range**	**${\text{h}}_{B}^{2}$^a^**
	**Y8679**	**J411**	**RIL population**		
SL	10.49	9.16	9.48	6.61–12.00	0.985
FSN	18.04	19.94	18.53	16.77–20.47	0.925
SSN	1.12	1.17	1.51	0.80–2.70	0.840
TSN	21.05	23.51	22.41	19.76–25.05	0.952
SC	1.82	2.32	2.15	1.61–2.82	0.979
PHT	75.85	86.60	91.73	58.42–108.28	0.976

a *Broad sense heritability based on a family mean basis was estimated across all nine environments*.

**Table 2:** **Correlation coefficients among the adjusted mean (BLUP) values of the traits spike length (SL), fertile spikelet number per spike (FSN), sterile spikelet number per spike (SSN), total spikelet number per spike (TSN), spikelet compactness (SC), and plant height (PHT)**.

**Trait**	**SL**	**FSN**	**SSN**	**TSN**	**SC**
FSN	0.19^**^				
SSN	0.50^**^	−0.01 NS			
TSN	0.42^**^	0.86^**^	0.49^**^		
SC	−0.90^**^	0.20^**^	−0.33^**^	0.00 NS	
PHT	0.29^**^	−0.16^*^	0.27^**^	0.00 NS	−0.33^**^

* and ** *indicate significance (2-tailed) at the 0.05 and 0.01 level, respectively; NS indicates not significant*.

### Linkage map construction

Genotyping of the Y8679/J411 RIL population with the wheat 90K SNP array resulted in 11,646 polymorphic markers that were used for the linkage analysis. In addition to the SNP markers, 215 SSR markers were utilized to genotype the Y8679/J411 population. After removing unlinked markers, the resulted map consisted of 10,990 markers (10,816 SNP markers and 174 SSR markers) mapped within 2034 unique loci, spanning 2875.3 cM in length with an average density of 1.4 cM/locus (Table 3). Of the 2034 unique loci, 334 (16.4%) had segregation ratios that deviated significantly (*P* < 0.05) from the expected 1:1 ratio (Table 3; Appendix B). Loci segregation distortion usually occurred in large contiguous blocks and chromosomes 3A, 4B, and 4D possessed large clusters of the most severely distorted loci (*P* < 0.00001). The 10,990 markers distributed unevenly on the 21 chromosomes, and the number ranged from 39 for chromosome 4D to 1344 for chromosome 1B. Eleven gaps (>20 cM) were observed on chromosomes 1A, 1D, 2A, 2D, 3B, 4A, 4B, 5A, 6A, and 7B (Figure S2).

**Table 3:** **The distribution and density of SSR and SNP markers on 21 wheat chromosomes**.

**Chromosome**	**SNP**	**SSR**	**Total markers**	**Unique locus**	**Length (cM)**	**cM/locus**	**Number of distorted locus (%)**
1A	498	0	498	92	135.9	1.5	8 (8.7)
2A	809	9	818	175	216.7	1.2	1 (0.6)
3A	470	1	471	116	138.0	1.2	78 (67.2)
4A	239	0	239	96	145.3	1.5	0 (0.0)
5A	463	8	471	111	158.9	1.4	0 (0.0)
6A^‡^	768	8	776	105	130.9	1.3	6 (5.7)
7A	650	16	666	173	222.6	1.3	3 (1.7)
1B	1327	17	1344	97	94.6	1.0	21 (21.6)
2B	1209	20	1229	209	194.1	0.9	11 (5.3)
3B	484	19	503	128	230.9	1.8	54 (42.2)
4B	351	9	360	73	186.9	2.6	51 (69.9)
5B	1230	8	1238	162	171.7	1.1	31 (19.1)
6B	778	13	791	130	192.7	1.5	28 (21.5)
7B	710	10	720	149	204.5	1.4	11 (7.4)
1D	205	3	208	25	80.6	3.2	0 (0.0)
2D	120	11	131	58	69.1	1.2	12 (20.7)
3D^‡^	187	10	197	66	133.5	2.0	10 (15.2)
4D^‡^	34	5	39	14	36.0	2.6	9 (64.3)
5D	72	0	72	13	6.3	0.5	0 (0.0)
6D	38	7	45	26	119.3	4.6	0 (0.0)
7D^‡^	174	0	174	16	7.0	0.4	0 (0.0)
A genome	3897	42	3939	868	1148.2	1.3	96 (11.1)
B genome	6089	96	6185	948	1275.2	1.4	207 (21.8)
D genome	830	36	866	218	451.9	2.1	31 (14.2)
Total	10816	174	10990	2034	2875.3	1.4	334 (16.4)

‡ *Chromosomes with two separated linkage groups. cM/locus was calculated by dividing their added genetic length by their added number of unique loci*.

Of 10,816 SNP-flanking sequences, the vast majority (87.82%) could be uniquely matched to wheat unigenes (Appendix A). Alignments between two or more neighboring SNPs to a single unigene were frequently observed. After removing duplicates, 5527 unigenes (with an average length of 1648.0 bp) were attached to the genetic linkage map. By comparing the sequences of 5527 unigenes, 3675 and 3685 orthologous genes from the *Brachypodium* and the rice genomes were uniquely tagged onto the genetic linkage map, respectively (Appendix A), providing an ideal resource for comparative analysis of targeted genomic regions.

### QTL mapping analysis

The QTLs that could be detected in four or more environments and in the combined analysis (BLUP) were regarded as “environmentally stable QTLs.” Among the detected 168 QTLs for spike morphological traits and PHT, 21 were environmentally stable QTLs. These 21 QTLs were located on nine chromosomes (1A, 1B, 2B, 2D, 3A, 4D, 5A, 7A, and 7B) (Appendix D). The rest 147 putative QTLs could only be detected in limited number of environments and are listed in Appendix E.

Thirty QTLs associated with SL were detected (Appendixes D, E). Six environmentally stable QTLs for SL were identified on chromosomes 1B, 2B, 2D, 5A, and 7B, and they were designated *QSl.cau-1B.2, QSl.cau-2B.2, QSl.cau-2D.2, QSl.cau-5A.4, QSl.cau-7B.1*, and *QSl.cau-7B.2*, respectively (Appendix D). Y8679 contributed effects for increased SL of the 1B, 2B, and 7B QTLs, which explained 4.88–7.96% of the SL variation for the combined analysis. J411 contributed effects for increased SL of the 2D and 5A QTLs, which explained as much as 35.55 and 15.25% of the SL variation for the combined analysis, respectively.

Among 31 QTLs associated with FSN, three environmentally stable QTLs were mapped on chromosomes 1A, 1B, and 3A, and they were designated *QFsn.cau-1A.4, QFsn.cau-1B.2*, and *QFsn.cau-3A.2*, respectively (Appendixes D, E). J411 contributed effects for increased FSN at the 1A and 3A loci. The 1A and 3A QTLs had LOD values of 7.50 and 3.38, and they explained 9.85 and 3.88% of the FSN variation for the combined analysis, respectively. Y8679 contributed effect for an increased FSN at the 1B locus, which explained as much as 28.78% of the FSN variation for the combined analysis.

Thirty QTLs were associated with SSN, most (21, 70.0%) of which were significant in one single environment (Appendixes D, E). The only two environmentally stable QTLs were identified on chromosomes 1B and 2D, and they were designated *QSsn.cau-1B.1* and *QSsn.cau-2D.2*, respectively. J411 contributed effect for a decreased SSN at the 1B locus, which had a LOD value of 8.22 and explained 18.22% of the SSN variation for the combined analysis. Y8679 contributed effect for a decreased SSN at the 2D locus. The 2D QTL was significant in all nine environments evaluated, and it had a LOD value of 12.63 and explained 17.13% of the SSN variation for the combined analysis.

Thirty-three significant QTLs were associated with TSN (Appendixes D, E), but the only two environmentally stable QTLs were identified on chromosomes 1B (*QTsn.cau-1B.2*) and 7A (*QTsn.cau-7A.3*). The LOD values of these loci ranged from 6.36 to 21.17, and they explained from 5.63 to 36.69% of the TSN variation for the combined analysis.

Thirty-three QTLs were found to be associated significantly with SC, five of which were environmentally stable QTLs (Appendixes D, E). These included one single QTL on chromosome 1A (*QSc.cau-1A.1*) and two QTLs each on chromosomes 2D (*QSc.cau-2D.1* and *QSc.cau-2D.2*) and 5A (*QSc.cau-5A.2* and *QSc.cau-5A.4*). J411 conferred effect for an increased SC at the 1A locus, and Y8679 contributed increasing alleles at the 2D and 5A loci. *QSc.cau-2D.2* was significant in all nine environments evaluated, and it had a LOD value of 20.34 and explained 26.27% of the SC variation for the combined analysis. Unlike those four loci on 2D and 5A, no coincidence of QTLs for SL, FSN, SSN, or TSN was detected at the 1A locus, suggesting that *QSc.cau-1A.1* controlled SC through a different mechanism.

Twelve QTLs associated with PHT were identified (Appendixes D, E). Three environmentally stable QTLs for PHT were detected on chromosomes 2D and 4D, and designated *QPht.cau-2D.1, QPht.cau-2D.2*, and *QPht.cau-4D.1*, respectively (Appendix D). Y8679 contributed effects for decreased PHT at all three loci, which explained 4.30–10.25% of the PHT variation for the combined analysis. *QPht.cau-2D.1* peaked at markers *Xcfd53* and *Xgwm261* (diagnostic marker for *Rht8*) on 2D indicating that the effects for decreased PHT for this locus was conferred by the dwarfing allele of *Rht8* from Y8679. *QPht.cau-4D.1* peaked at marker *Xgwm165*, which is closely linked with the centromere of chromosome 4D. Since no linkage group was mapped for the short arm of chromosome 4D, it is necessary to further clarify the relationship between *QPht.cau-4D.1* and *Rht-D1*.

### Comparative analysis of genomic regions harboring stable QTLs for spike morphological traits

**Figure 3:**
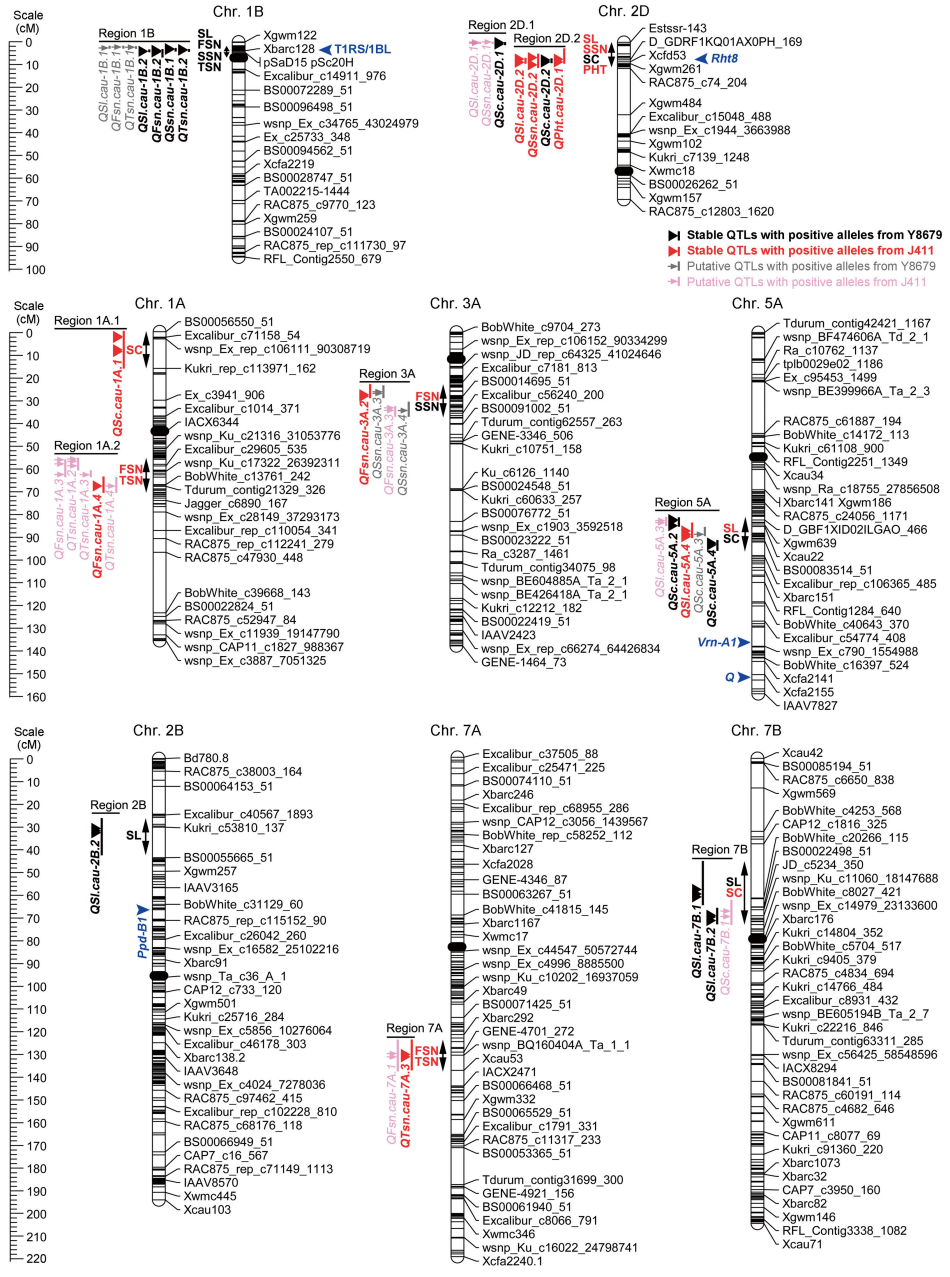
**Chromosomal locations of 10 genomic regions associated with spike morphological traits**. Only environmentally stable QTLs and their tightly-linked putative QTLs are shown. A centiMorgan (cM) scale is shown on the left. Black ellipses represent the approximate locations of the centromeres. Vertical bars represent the LOD-2 confidence interval for the location of each QTL. Black and red triangles indicate environmentally stable QTLs with positive alleles from parent Y8679 and parent J411, respectively. Gray and pink arrows indicate putative QTLs with positive alleles from parent Y8679 and parent J411, respectively. Double headed arrows indicate the genomic regions characterized by QTLs or QTL clusters. Traits alongside the double headed arrows are spike length (SL), fertile spikelet number per spike (FSN), sterile spikelet number per spike (SSN), total spikelet number per spike (TSN), spikelet compactness (SC), and plant height (PHT). The known positions of the 1RS/1BL translocation (T1RS/1BL), *Rht8, Ppd-B1, Vrn-A1*, and *Q* loci are presented in blue arrows (Korzun et al., 1998; Liu et al., 2008; Cavanagh et al., 2013; Wang et al., 2014; Faris et al., 2014a).

Ten genomic regions covering 18 stable QTLs for spike morphological traits are listed in Table 4 and are shown in Figure 3. In addition, putative QTLs for SL, FSN, SSN, TSN, and SC mapped within these 10 genomic regions were also shown in Figure 3. Regions 1A.1 and 2B mapped a single QTL for SC (*QSc.cau-1A.1*) and SL (*QSl.cau-2B.2*), respectively. The rest eight genomic regions contained 32 co-localized QTLs (16 stable QTLs and 16 putative QTLs) with individual genomic region harboring QTLs for two to four traits. These co-localized QTLs shared confidence intervals and had tightly linked QTL peak positions (usually within 10 cM), which are indicative of potential pleiotropy among the traits.

**Table 4:** **The 10 genomic regions harboring environmentally stable QTLs for spike morphological traits in the Y8679/J411 RIL population**.

**Genomic region**	**Interval (cM)**	**Associated traits^a^**	**Included QTLs^b^**	**Detected environment^c^**	**References**
Region 1A.1	0.00–16.10	SC (−)	***QSc.cau-1A.1***	E2/E3/E5/E6/E7/E9/C	
Region 1A.2	55.40–71.40	FSN (−)	*QFsn.cau-1A.3*; ***QFsn.cau-1A.4***	E2/E3/E6/E9/C	Heidari et al., 2011
		TSN (−)	*QTsn.cau-1A.2*; *QTsn.cau-1A.3*; *QTsn.cau-1A.4*	E2/E3/E6/E7/C	
Region 1B	2.00–7.10	SL (+)	*QSl.cau-1B.1*; ***QSl.cau-1B.2***	E1/E3/E7/E9/C	Gao et al., 2015
		FSN (+)	*QFsn.cau-1B.1*; ***QFsn.cau-1B.2***	E1/E2/E3/E5/E7/E8/E9/C	
		SSN (+)	***QSsn.cau-1B.1***	E2/E4/E5/E6/C	Cui et al., 2012
		TSN (+)	*QTsn.cau-1B.1*; ***QTsn.cau-1B.2***	E1/E2/E3/E4/E5/E6/E7/E8/E9/C	
Region 2B	26.50–43.20	SL (+)	***QSl.cau-2B.2***	E2/E4/E5/E6/E9/C	Xu et al., 2014
Region 2D.1	0.00–1.30	SL (−)	*QSl.cau-2D.1*	E2/E4/E9	
		SSN (−)	*QSsn.cau-2D.1*	E3/C	
		SC (+)	***QSc.cau-2D.1***	E1/E2/E6/E8/E9/C	
Region 2D.2	5.10–11.10	SL (−)	***QSl.cau-2D.2***	E1/E2/E3/E4/E5/E6/E7/E8/E9/C	Xu et al., 2014
		SSN (−)	***QSsn.cau-2D.2***	E1/E2/E3/E4/E5/E6/E7/E8/E9/C	Xu et al., 2014
		SC (+)	***QSc.cau-2D.2***	E1/E2/E3/E4/E5/E6/E7/E8/E9/C	Heidari et al., 2011
		PHT(−)	***QPht.cau-2D.1***	E1/E3/E5/E8/C	Xu et al., 2014
Region 3A	23.20–37.50	FSN (−)	***QFsn.cau-3A.2***; *QFsn.cau-3A.3*	E3/E5/E6/E7/C	Xu et al., 2014
		SSN (+)	*QSsn.cau-3A.3*; *QSsn.cau-3A.4*	E1/E3/E7	
Region 5A	80.80–95.40	SL (−)	*QSl.cau-5A.3*; ***QSl.cau-5A.4***	E2/E4/E5/E6/E7/E8/E9/C	Yu et al., 2014
		SC (+)	***QSc.cau-5A.2***; *QSc.cau-5A.3*; ***QSc.cau-5A.4***	E1/E2/E4/E5/E6/E7/E8/E9/C	Xu et al., 2014
Region 7A	123.50–137.50	TSN (−)	***QTsn.cau-7A.3***	E1/E3/E6/E7/E9/C	Xu et al., 2014
		FSN (−)	*QFsn.cau-7A.1*	E5/E6/E9/C	Xu et al., 2014
Region 7B	44.60–73.10	SL (+)	***QSl.cau-7B.1***; ***QSl.cau-7B.2***	E2/E3/E4/E6/E9/C	
		SC (−)	*QSc.cau-7B.1*	E1/E2	

a *Traits are spike length (SL), fertile spikelet number per spike (FSN), sterile spikelet number per spike (SSN), total spikelet number per spike (TSN), spikelet compactness (SC), and plant height (PHT). The plus (“+”) and minus (“−”) signs within the brackets indicate Y8679 and J411 contributed increasing alleles, respectively*.

b *The QTLs shown in bold are environmentally stable QTLs*.

c *C indicates the combined QTL analysis based on the BLUP values across nine environments*.

**Figure 4:**
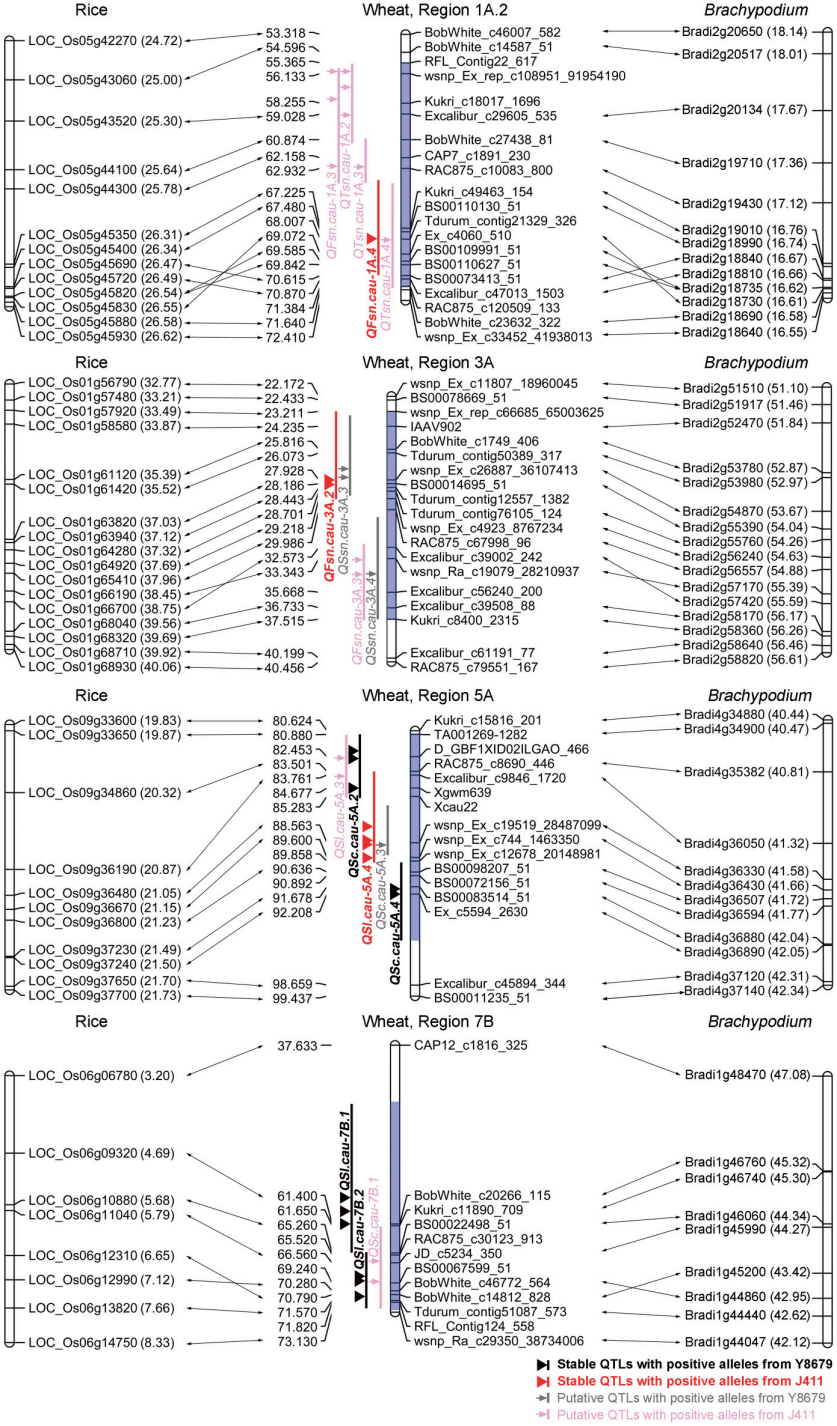
**Comparative mapping results of four genomic regions harboring stable QTLs for spike morphological traits**. The number in brackets indicates the physical position (Mb) of rice and *Brachypodium* genes. Blue shadow indicates the range of the targeted genomic region.

The protein sequences of rice and *Brachypodium* showing best hits to SNP markers mapped within nine genomic regions (excluding Region 1B, T1RS/1BL) were further analyzed for a comparative mapping purpose. Functional predictions of these rice and *Brachypodium* proteins were obtained from PGSB database (http://pgsb.helmholtz-muenchen.de/plant/index.jsp, verified 7 September 2016) and are listed in Appendix F. Regions 1A.1, 2B, 2D.1, 2D.2, and 7A exhibit indeterminate collinearities with rice and *Brachypodium* genomes (Table S2). Regions 1A.2, 3A, 5A, and 7B are highly syntenic to rice chromosomes 5 (25.00–26.58 Mb), 1 (33.21–39.69 Mb), 9 (19.87–21.70 Mb), and 6 (3.20–8.33 Mb), respectively, and to *Brachypodium* chromosomes 2 (16.58–18.01 Mb), 2 (51.46–56.26 Mb), 4 (40.47–42.31 Mb), and 1 (42.12–47.08 Mb), respectively (Figure 4; Table S2).

### Validation of the consequences of 1RS/1BL translocation and *Rht8*

The 5.5 cM interval on chromosome 1BS mapped 1031 SNP markers and 13 SSR markers, and hence we deduced that one of the parents might carry the 1RS/1BL translocation. Thus, two 1RS specific markers (*pSaD15* and *pSc20H*; Liu et al., 2008) were used for analysis (Figure S3). The results showed that 75 RILs carried the 1RS/1BL translocation donated by parent Y8679, which exhibited significant (*P* < 0.05) segregation distortion with approximately 61% J411 alleles. Similar segregation ratios have also been reported in other studies in RIL lines from crosses between 1B and 1RS/1BL parents, suggesting biased transmission against gametes carrying the translocation (Mathews et al., 2008; McIntyre et al., 2010). Adjusted mean (BLUP) values across nine environments were used to determine differences between 1RS/1BL and 1B lines in SL, FSN, SSN, TSN, and SC (Table 5). No significant variation was detected between 1RS/1BL and 1B lines for SC. Compared with 1B lines, the 1RS/1BL lines had higher SL (7.2%), FSN (4.2%), SSN (24.8%), and TSN (5.9%). Consequently, four major and environmentally stable QTLs were detected at the *pSaD15* locus, and they had LOD values from 3.33 to 21.17 and explained from 6.74 to 36.69% of the variations for the combined analysis (Figures 3; Figure S4; Appendix D). The 1RS/1BL translocation from Y8679 was associated with increased SL, FSN, SSN, and TSN.

**Table 5:** **Variations between 1RS/1BL and 1B lines for spike length (SL), fertile spikelet number per spike (FSN), sterile spikelet number per spike (SSN), total spikelet number per spike (TSN), and spikelet compactness (SC)**.

**Trait**	**1RS/1BL lines**	**1B lines**	***p*-value**
SL	9.87 ± 0.93	9.21 ± 0.96	3.72E-06
FSN	18.98 ± 0.69	18.22 ± 0.68	1.22E-12
SSN	1.71 ± 0.41	1.37 ± 0.31	9.45E-10
TSN	23.18 ± 0.87	21.89 ± 0.76	1.86E-21
SC	2.13 ± 0.20	2.16 ± 0.22	0.40

Four co-localized stable QTLs for SL, SSN, SC, and PHT overlapped with the semi-dwarfing gene *Rht8*, which raises the possibility that *Rht8* conferred pleiotropic effects on these traits. Therefore, one commonly used diagnostic marker for *Rht8, Xgwm261*, was selected for investigation (Korzun et al., 1998). Of the 191 RILs, 96 and 90 RILs amplified the 192 bp (*Rht8c* allele from parent Y8679) and 174 bp (*Rht8b* allele from parent J411) fragments, respectively (Figure S5). In addition, five RILs amplified smeared or fuzzy products when visualized with silver staining. Adjusted mean (BLUP) values across nine environments were used to identify differences between *Rht8c* and *Rht8b* lines in SL, FSN, SSN, TSN, SC, and PHT (Table 6). The *Rht8c* allele was associated with significant reductions (*P* < 0.05) in SL (11.1%), SSN (20.7%), TSN (2.2%), and PHT (3.3%), and with a remarkable increase in SC (10.3%). Since no significant effect was detected for the *Rht8c* allele on FSN (0.0%, *p* = 0.97), the slight reduction in TSN was probably due to reduction in SSN.

**Table 6:** **Variations between ***Rht8c*** and ***Rht8b*** lines for spike length (SL), fertile spikelet number per spike (FSN), sterile spikelet number per spike (SSN), total spikelet number per spike (TSN), spikelet compactness (SC), and plant height (PHT)**.

**Trait**	***Rht8c***	***Rht8b***	***p*-value**
SL	8.93 ± 0.87	10.04 ± 0.79	1.63E-16
FSN	18.54 ± 0.76	18.54 ± 0.80	0.97
SSN	1.34 ± 0.31	1.69 ± 0.39	1.69E-10
TSN	22.19 ± 0.94	22.68 ± 1.06	9.81E-04
SC	2.25 ± 0.21	2.04 ± 0.15	1.84E-13
PHT	90.21± 9.67	93.29 ± 7.96	0.02

## Discussion

### Comparison between genetic linkage maps constructed with the iSelect 9K and 90K SNP arrays

The prerequisite of genetic studies is constructing a high-quality, saturated genetic map. The recently developed iSelect 9K and 90K arrays (Cavanagh et al., 2013; Wang et al., 2014) designed to characterize genetic variation in hexaploid wheat populations have extensive applications in QTL mapping (Wu et al., 2015b), genome-wide association studies (GWAS) (Gao et al., 2016), genomic selection (He et al., 2016), and establishment of heterotic groups (Zhao et al., 2015). In the present study, we constructed a dense genetic linkage map for the Y8679/J411 RIL population, using 10,816 polymorphic SNP markers from an iSelect 90K array and 174 polymorphic SSR markers. Of the 10,816 SNP markers, 9564 (88.4%) were mapped by Wang et al. (2014) and Gao et al. (2015). Remarkably, 1,252 (11.6%) SNP markers were newly mapped in this study (Appendix A). The order of SNP markers along the chromosomes was basically consistent with Wang et al. (2014). The genetic length of this map was 2875.3 cM, similar to reported maps in hexaploid wheat (Somers et al., 2004; Wu et al., 2015b). Notably, the high number (11,646) of polymorphic SNP markers between Y8679 and J411 is comparable to Jin et al. (2016) and Perez-Lara et al. (2016), who detected 12,205 and 10,342 polymorphic SNPs between two parental lines, respectively. Collectively, these data suggest that the iSelect 90K array is a powerful tool for genotyping analysis in hexaploid wheat.

In our previous study, we genotyped the Y8679/J411 RIL population using the iSelect 9K array and constructed a SNP-based genetic linkage map (Zhai et al., 2015). Regrettably, 10 huge gaps over 20 cM existed on six chromosomes and the genetic coverage of D genome was gravely inadequate. For example, chromosomes 4D and 7D covered only 2.9 and 2.6 cM in length, respectively, while chromosome 5D mapped no linkage group. Hence, the iSelect 90K array used in the present study was anticipated to fill the gaps and enlarge the genetic coverage of the D genome. As compared with map constructed with iSelect 9K array, the genetic coverage, the total number of SNP markers and unique loci in the present study increased by 73.2, 469.0, and 103.4%, respectively, and the average intervals between unique loci (loci/cM) decreased by 17.6% (Table S3). Notably, although some huge gaps (>20 cM) persisted, 5 of the 10 gaps were successfully narrowed. However, no substantial improvement was observed for the D genome, especially for chromosomes 5D (6.3 cM) and 7D (7 cM), which may hinder the detection of possible QTLs on these chromosomes. Collectively, these data indicated that both the iSelect 9K and 90K arrays have considerable limitations when dealing with the D genome. Therefore, studies focusing on chromosomes from the D genome are recommended to use much larger genotyping platforms, such as the recently developed 660K SNP chip (Jin et al., 2016).

### Novel QTLs identified for wheat spike morphology on chromosomes 1AS, 2DS, and 7BS

In order to enhance the yield potential of wheat, breeders have tried to alter the sink capacity through modifying the spike morphology of wheat (Reynolds et al., 1999). The present study identified 10 genomic regions on eight chromosomes, which harbor 18 environmentally stable QTLs for five spike morphological traits (SL, FSN, SSN, TSN, and SC). Seven genomic regions on chromosomes 1AL, 1BS, 2BS, 2DS, 3AL, 5AL, and 7AS coincide with previously reported QTLs/genes. For example, Region 1A.2 (*QFsn.cau-1A.4*) corresponds to reported QTLs for spikelet number (Heidari et al., 2011; Wang et al., 2011). Regions 2B (*QSl.cau-2B.2*), 3A (*QFsn.cau-3A.2*), and 7A (*QTsn.cau-7A.3*) have been detected by Xu et al. (2014) using the Xiaoyan 54/Jing 411 RIL population, which shared one common parental line (Jing 411) with our population. Region 7A (*QTsn.cau-7A.3*) locates in a region similar to gene *TaMOC1-7A*, which was associated with spikelet number per spike in common wheat (Zhang et al., 2015). Besides, three novel genomic regions including Regions 1A.1 (*QSc.cau-1A.1*), 2D.1 (*QSc.cau-2D.1*), and 7B (*QSl.cau-7B.1* and *QSl.cau-7B.2*) are firstly presented in this study.

A QTL on chromosome 1AS controlling SC (*QSc.cau-1A.1*) has not been reported previously, indicating that this novel QTL could be due to specific genetic materials used in the present study. Nevertheless, previous studies have mapped QTLs for spikelet number or spike length at the same chromosomal region (Kumar et al., 2007; Ma et al., 2007). However, no association of the *QSc.cau-1A.1* locus with spike length or spikelet number was detected under all environments in this study, suggesting that *QSc.cau-1A.1* is most likely different from reported QTLs for spike length or spikelet number on chromosome 1AS. Two neighboring QTLs on chromosome 7BS, *QSl.cau-7B.1*, and *QSl.cau-7B.2*, were detected in 4–5 environments. To the best of our knowledge, no QTL for spike length has been detected on the short arm of chromosome 7B. Therefore, these two QTLs detected in our RIL population represent two novel loci controlling spike length in wheat.

A novel locus, *QSc.cau-2D.1*, distal to *Rht8* on the short arm of chromosome 2D has not been reported in previous studies. Co-localized QTLs for SL and SSN at the *QSc.cau-2D.1* locus were also detected in our RIL population, explaining 13.77–18.45 and 7.88–8.90% of the phenotypic variation, respectively (Figure 3; Appendix E). Since *QSc.cau-2D.1* is only 8.7 cM away from *QSc.cau-2D.2*, a major QTL for SC with the strongest effect, it is difficult to conclude whether *QSc.cau-2D.1* is a shadow or genuine QTL for SC. Fortunately, a RIL line (YJ-171) from our population exhibited residual heterozygosity at both the *QSc.cau-2D.1* and *QSc.cau-2D.2* loci (data not shown). A mapping population deriving from YJ-171 has been developed for dissection of these two QTLs, and preliminary result supports that *QSc.cau-2D.1* is a genuine QTL controlling spike length and spikelet compactness (unpublished data). Therefore, it seems that the *QSc.cau-2D.1* locus identified in our population represents a novel locus for spike length and spikelet compactness in wheat.

### Pleiotropic QTLs on chromosomes 2DS and 5AL

The *QPht.cau-2D.1* locus on the short arm of chromosome 2D was identical to gene *Rht8* (Korzun et al., 1998). This locus has been associated with QTLs for plant height, spike length, spikelet number, spikelet compactness, thousand grain weight, and grain yield (Ma et al., 2007; Cui et al., 2012; Xu et al., 2014; Wang Y. S. et al., 2015). In this study, The *QPht.cau-2D.1* locus was detected to have pleiotropic effects for SL, SSN, SC, and PHT. The Y8679 derived allele decreased SL and SSN, but increased SC under all evaluated environments. Our results support the recent findings that the *Rht8c* introgression decreased spike length with constant spikelet number, resulting in a semi-compacted spike morphology (Wu et al., 2014; Kowalski et al., 2016), but contrasting with other recent studies showing that the *Rht8c* allele has no significant effect on spike length (Gasperini et al., 2012; Rebetzke et al., 2012).

The *QSl.cau-5A.4* locus co-localized with two neighboring QTLs for SC (*QSc.cau-5A.2* and *QSc.cau-5A.4*) in this study. The *QSl.cau-5A.4* locus has been associated with QTLs for spike length (Kumar et al., 2007; Cui et al., 2012; Yu et al., 2014; Wu et al., 2015a), spikelet number (Kumar et al., 2007; Ding et al., 2011; Cui et al., 2012) and spikelet compactness (Jantasuriyarat et al., 2004; Katkout et al., 2014; Xu et al., 2014) in many studies. Moreover, the *QSl.cau-5A.4* locus was different from *Vrn-A1* and *Q* (Figure 3), which deserves for further investigation.

### Genotype-dependent effects of the 1RS/1BL translocation on spike morphology in wheat

To date, a number of studies have shown that the higher yield potential of the 1RS/1BL lines is caused by the higher kernel number per spike, which can be attributed to the higher spikelet number per spike (Schlegel and Meinel, 1994; Villareal et al., 1998; Zhao et al., 2012). Consistent with these data, our results showed that the 1RS/1BL translocation increased the FSN and TSN in nearly all environments (Appendix D). We also found that the 1RS/1BL translocation had a considerable positive effect on the SSN and SL, which is consistent with the results of previous studies (Cui et al., 2012; Gao et al., 2015). QTLs were not detected for SC in any of the nine individual environments, suggesting that the 1RS/1BL translocation has no significant influence on spikelet compactness, which is inconsistent with the results of Tahmasebi et al. (2015). Moreover, other studies have also shown a loose connection between the 1RS/1BL translocation and spike morphological traits (Villareal et al., 1995; Griffiths et al., 2015). Collectively, these data demonstrate that the impact of the 1RS/1BL translocation on performance is highly linked to the plant's genetic background and environmental conditions, which limit the use of 1RS/1BL translocation lines as a source of genetic variation. Thus, it will be necessary to explore and utilize novel genetic diversity for super high-yield wheat breeding.

### Candidate genes controlling wheat spike morphology

The primary utility of gene-based linkage maps is for comparative mapping, which has become a tool for comparing gene order and content across related grass species (Somyong et al., 2011). A number of successful studies have reported the fine mapping of QTLs for agronomically important traits based on orthologous regions across several grass species (Chen et al., 2007; Handa et al., 2008; Somyong et al., 2011). In the present study, we report integration of the SNP-based genetic linkage map with 3,675 *Brachypodium* genes and 3,685 rice genes, which is anticipated to assist the comparative mapping of detected QTLs. Of 10 genomic regions harboring stable QTLs, Region 1B was not considered for comparative analysis because of the 1RS/1BL translocation. Five genomic regions have indeterminate collinearities with model genomes owing to two reasons, i.e., limited density of polymorphic SNP markers (Regions 2D.1, 2D.2, and 7A) and chaotic collinearity with multiple chromosomes from model genomes (Regions 1A.1 and 2B). Remarkably, four genomic regions harboring stable QTLs for SL (Regions 5A and 7B), FSN (Regions 1A.2 and 3A) and SC (Region 5A) exhibit good collinearity with model genomes (Figure 4). Region 3A contains two neighboring QTLs for FSN, i.e., one environmentally stable QTL (*QFsn.cau-3A.2*) and one putative QTL (*QFsn.cau-3A.3*). Comparative analysis demonstrated that the peak regions of *QFsn.cau-3A.2* and *QFsn.cau-3A.3* are syntenic to rice chromosome 1 at 35.52–37.12 and 38.45–39.56 Mb, respectively (Figure 4). Interestingly, rice chromosome 1 harbors the two cloned genes *LAX1* (*LOC_Os01g61480*) and *EG1* (*LOC_Os01g67430*) governing the spikelet development, which are located at 35.56 and 39.18 Mb, respectively (Komatsu et al., 2001; Li et al., 2009). These results suggest that the two QTLs for FSN identified on Region 3A could be orthologous to *LAX1* or *EG1*, which deserve further investigation.

## Author contributions

ZN and QS conceived the project; SX developed the Yumai 8679/Jing 411 RIL population; HZ, ZF, and JL carried out experiments; XL performed bioinformatics analysis; HZ analyzed experimental results; HZ, ZN, and QS wrote the manuscript.

### Conflict of interest statement

The authors declare that the research was conducted in the absence of any commercial or financial relationships that could be construed as a potential conflict of interest.

## Abbreviations

FSN: Fertile spikelet number per spike
PHT: Plant height
QTL: Quantitative trait locus
RIL: Recombinant inbred line
SC: Spikelet compactness
SL: Spike length
SNP: Single nucleotide polymorphism
SSN: Sterile spikelet number per spike
SSR: Simple sequence repeat
TSN: Total spikelet number per spike.
