# Supplementary material for: QTL Analysis of Spike Morphological Traits and Plant Height in Winter Wheat (Triticum aestivum L.) Using a High-Density SNP and SSR-Based Linkage Map
Source: Front Plant Sci. 2016 Nov 7;7:1617. doi: 10.3389/fpls.2016.01617 (PMC5097907; doi:10.3389/fpls.2016.01617)
Supplement: Supplementary file 2 [file DataSheet2.pdf]

Supporting Information: Zhai et al. 10.3389/fpls.2016.01617

QTL Analysis of Spike Morphological Traits and Plant Height in Winter Wheat (*Triticum aestivum* L.) Using a High-Density SNP and SSR-Based Linkage Map

Huijie Zhai [1, 2], Zhiyu Feng [1, 2], Jiang Li [1, 2], Xinye Liu [1, 2], Shihe Xiao [3], Zhongfu Ni [1, 2 *], Qixin Sun [1, 2 *]

[1] State Key Laboratory for Agrobiotechnology, Key Laboratory of Crop Heterosis and Utilization, Beijing Key Laboratory of Crop Genetic Improvement, China Agricultural University, Beijing, China, [2] National Plant Gene Research Centre, Beijing, China, [3] Institute of Crop Science, Chinese Academy of Agricultural Sciences, Beijing, China

***Correspondence:**
Zhongfu Ni, nizf@cau.edu.cn; Qixin Sun, qxsun@cau.edu.cn

Table S1 Summary of climate data across all nine environments

Location, year	Code	Latitude, longitude	Soil type	Planting date	Harvesting date	Average min temp. (℃)		Average max temp. (℃)		Days max temp. >30 ℃		Precipitation (mm) [a]		Generation of the 191 RILs
						Pre-anthesis	Post-anthesis	Pre-anthesis	Post-anthesis	Pre-anthesis	Post-anthesis	Pre-anthesis	Post-anthesis	
Anhui, 2011-2012	E2	33°48' N, 116°35' E	Lime concretion black soil	2011/10/15	2012/6/4	3.2	17.6	11.4	28.5	0	7	208.1	30.1	F_{10}
Anhui, 2012-2013	E5			2012/10/14	2013/6/11	4.2	18.4	13.3	28.8	2	15	163.5	183.7	F_{11}
Shaanxi, 2011-2012	E4	34°17' N, 108°04' E	Silty clay loam	2011/10/8	2012/6/15	2.6	15.8	11.4	28.1	0	10	269.3	64.0	F_{10}
Shaanxi, 2012-2013	E7			2012/10/6	2013/6/8	2.0	16.1	12.8	27.6	2	13	52.7	176.1	F_{11}
Hebei, 2014-2015	E9	37°56' N, 114°42' E	Calcareous sandy loam	2014/10/2	2015/6/12	1.8	17.9	13.1	29.7	2	16	-	-	F_{12}
Beijing, 2010-2011	E1	40°08' N, 116°10' E	Calcareous sandy loam	2010/10/2	2011/6/10	-1.5	15.3	10.6	29.2	1	13	89.0	36.9	F_{9}
Beijing, 2011-2012	E3			2011/10/1	2012/6/11	0.0	16.6	10.1	29.1	2	11	115.6	36.3	F_{10}
Beijing, 2012-2013	E6			2012/10/3	2013/6/13	-0.9	16.9	9.6	28.3	0	5	140.5	21.1	F_{11}
Beijing, 2014-2015	E8			2014/10/3	2015/6/9	1.5	17.2	12.5	29.6	0	17	-	-	F_{12}

[a] The precipitation information of E8 and E9 was not recorded

Table S2 Comparative analysis of nine genomic regions harboring stable QTLs for spike morphological traits

Genomic region	Interval (cM)	Rice			*Brachypodium*		
		Chr.	Interval (Mb)	Collinearity	Chr.	Interval (Mb)	Collinearity
Region 1A.1	0.00-16.10	Chr.5	?	Poor	Chr.2	?	Poor
Region 1A.2	55.40-71.40	Chr.5	25.00-26.58	Good	Chr.2	16.58-18.01	Good
Region 2B	26.50-43.20	?	?	Poor	?	?	Poor
Region 2D.1	0.00-1.30	Chr.4	?-0.20	Poor	Chr.5	?-2.52	Poor
Region 2D.2	5.10-11.10	?	?	Poor	Chr.5	3.15-6.61	Poor
Region 3A	23.20-37.50	Chr.1	33.21-39.69	Good	Chr.2	51.46-56.26	Good
Region 5A	80.80-95.40	Chr.9	19.87-21.70	Good	Chr.4	40.47-42.31	Good
Region 7A	123.50-137.50	?	?	Poor	?	?	Poor
Region 7B	44.60-73.10	Chr.6	3.20-8.33	Good	Chr.1	42.12-47.08	Good

“?” indicates uncertainty of collinearity between wheat and model genomes at corresponding genomic regions

Table S3 Comparison between genetic linkage maps constructed with the iSelect 9K and 90K SNP arrays

Genome	SNP array [a]	SNP	Unique loci	Length (cM)	cM/loci
A genome	9K	899	433	719.1	1.7
	90K	3897	868	1148.2	1.3
	Delta	333.5%	100.5%	59.7%	-23.5%
B genome	9K	926	484	703.5	1.5
	90K	6089	948	1275.2	1.4
	Delta	557.6%	95.9%	81.3%	-6.7%
D genome	90K	76	83	237.3	2.9
	9K	830	218	451.9	2.1
	Delta	992.1%	162.7%	90.4%	-27.6%
Total	9K	1901	1000	1659.9	1.7
	90K	10816	2034	2875.3	1.4
	Delta	469.0%	103.4%	73.2%	-17.6%

[a] Detailed information of genetic linkage map construction using iSelect 9K array can be accessed in our previous study (Zhai et al. 2015)

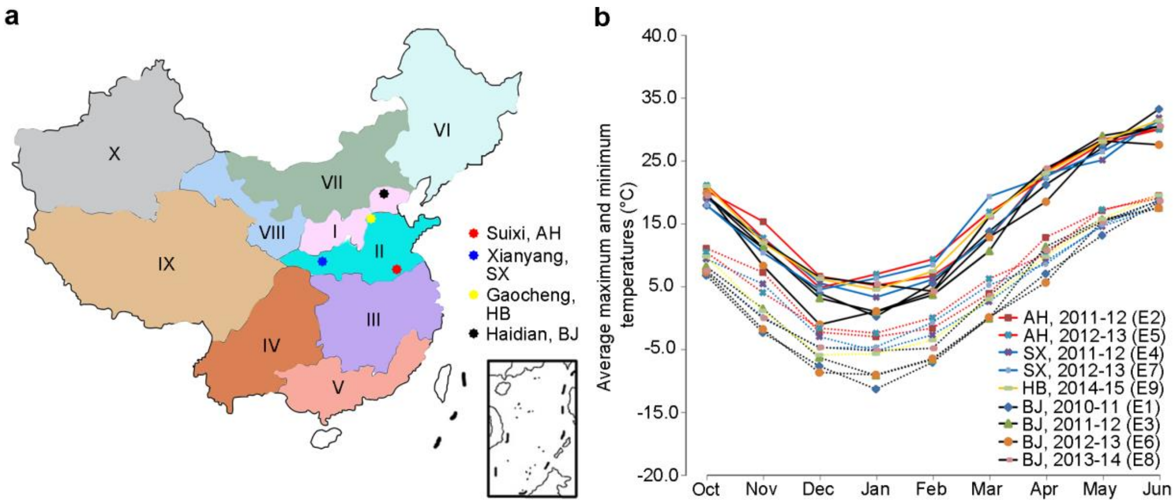

Figure S1 Experimental sites and temperature summary across nine environments. (**a**), The four locations selected for evaluation of the Y8679/J411 RIL population. These four locations are representative areas of two crucial wheat production zones, Northern Winter Wheat Zone (I) and Yellow and Huai River Valleys Facultative Wheat Zone (II), which produce 68% of the current total wheat production in China (Wang et al. 2009). The rest wheat production zones are Middle and Lower Yangtze Valleys Autumn-Sown Spring Wheat Zone (III), Southwestern Autumn-Sown Spring Wheat Zone (IV), Southern Autumn-Sown Spring Wheat Zone (V), Northeastern Spring Wheat Zone (VI), Northern Spring Wheat Zone (VII), Northwestern Spring Wheat Zone (VIII), Qinghai-Tibetan Plateau Spring-Winter Wheat Zone (IX) and Xinjiang Winter-Spring Wheat Zone (X). (**b**), Monthly average maximum and minimum temperatures across nine environments. Solid and dashed lines indicate the average maximum and minimum temperatures, respectively.

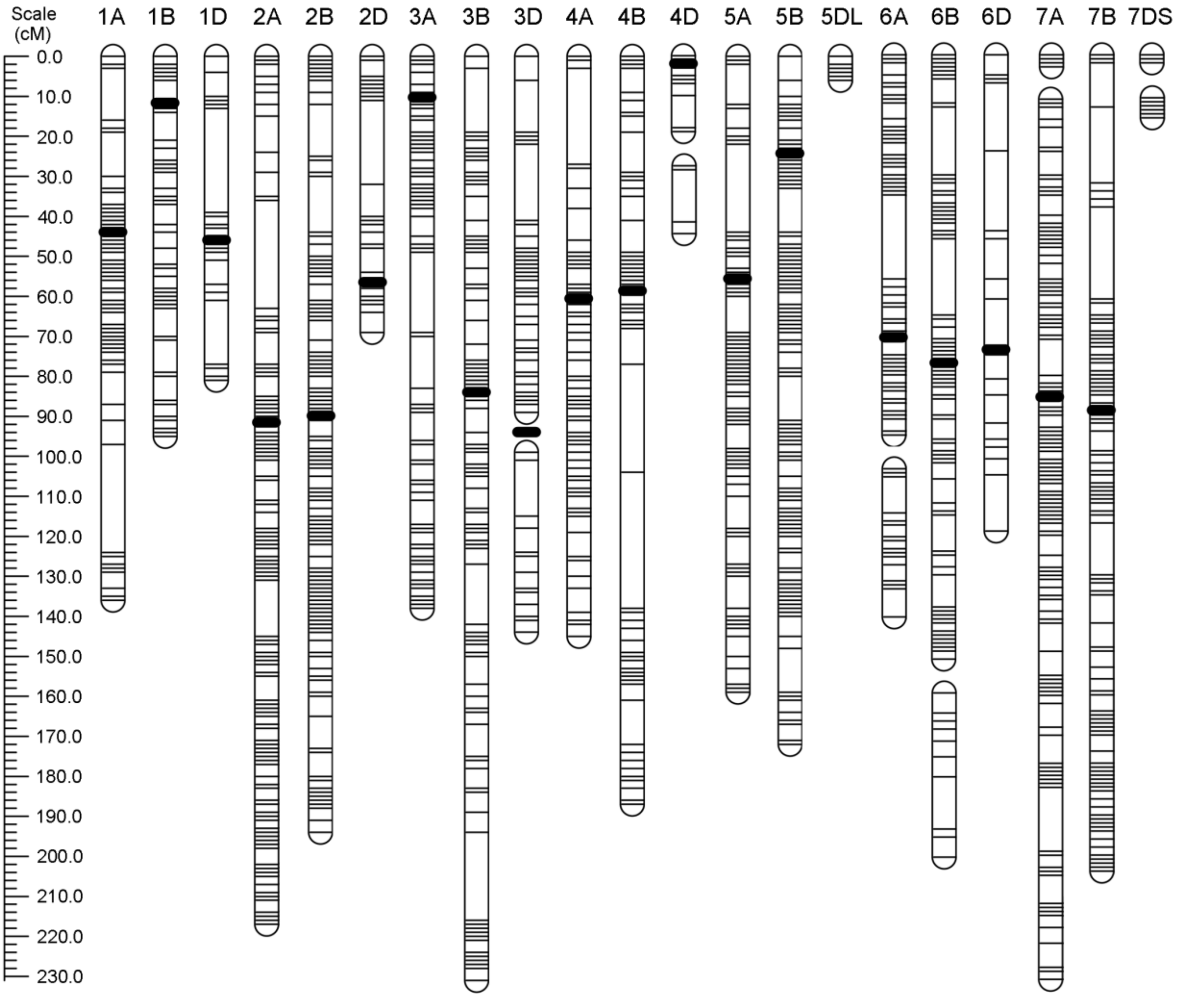

Figure S2 Illustration of 21 wheat chromosomes in the Y8679/J411 population. A centiMorgan (cM) scale is shown on the left. Approximate locations for centromeres are shown in black ellipses. Centromeres of chromosomes 5D and 7D are not shown because both chromosomes mapped only one arm.

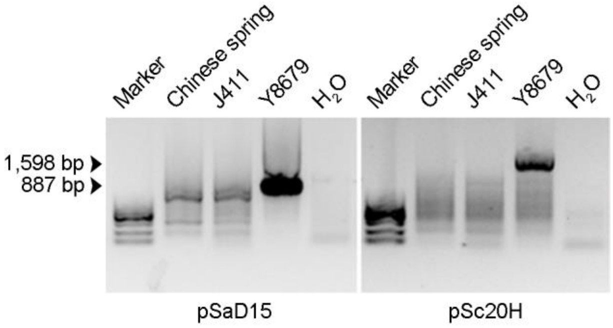

Figure S3 Amplified polymorphisms of two 1RS specific markers between parent Y8679 and parent J411. pSaD15 and pSc20H amplified two clear 887-bp and 1,598-bp fragments in parent Y8679, respectively. Chinese spring and water were used as negative controls.

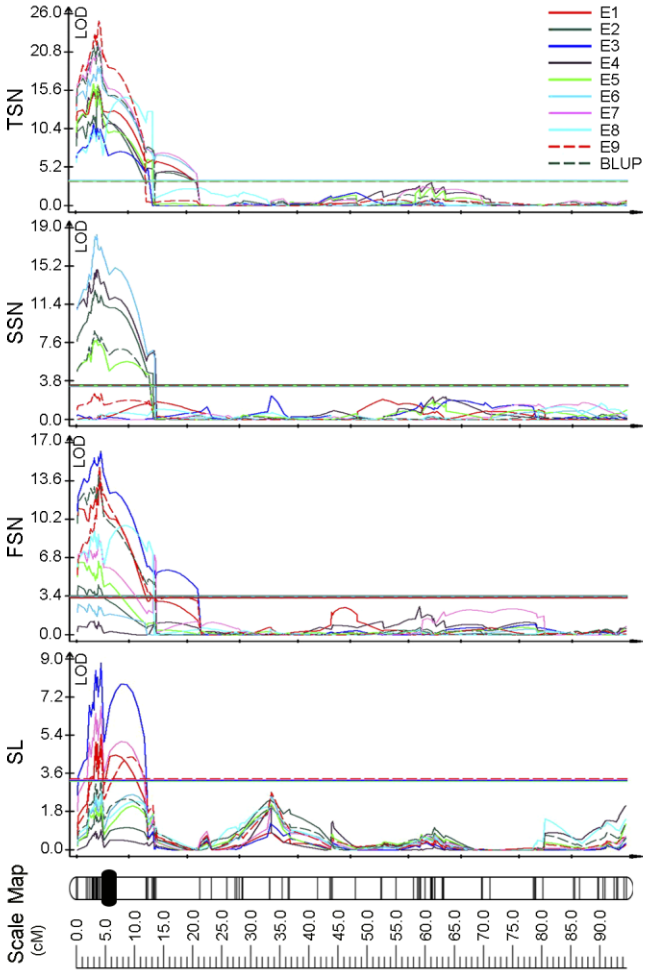

Figure S4 Co-localization of the QTLs for spike length (SL), fertile spikelet number per spike (FSN), sterile spikelet number per spike (SSN) and total spikelet number per spike (TSN) on chromosome 1B across multiple environments

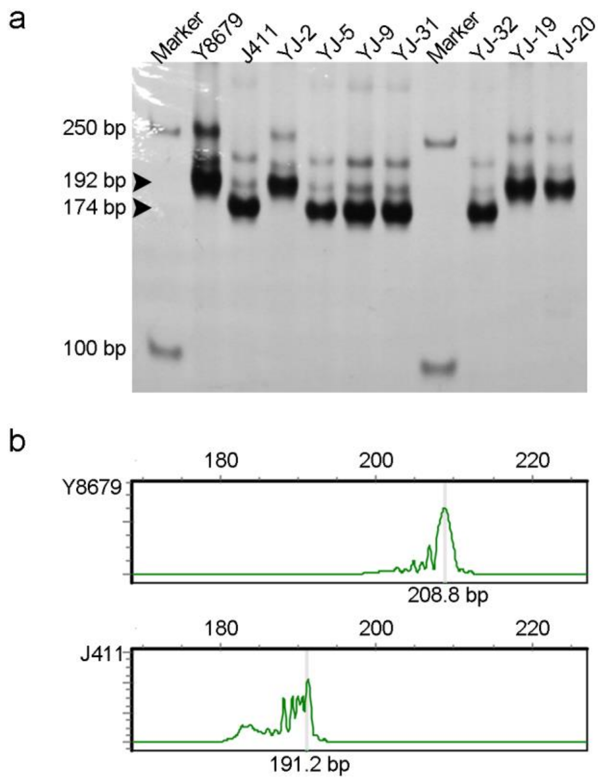

Figure S5 Amplified polymorphism of *Xgwm261* between parent Y8679 and parent J411. (a) Amplified products visualized by silver staining; (b) Amplified products visualized by ABI DNA Analyzer. A M13-tail (5'-ACGACGTTGTAAAACGAC-3') of 18 bp was added to the 5'-end of the forward primer.
